# Comparison of Intravitreal Bevacizumab and Intravitreal Diclofenac in the Treatment of Diabetic Macular Edema: a 6-month Follow-up

**Published:** 2017

**Authors:** Hooshang FAGHIHI, Hanif YAHYAPOUR, Raziyeh MAHMOUDZADEH, Shahin FAGHIHI

**Affiliations:** 1 Farabi Eye Hospital, Tehran University of Medical Sciences, Tehran, Iran

**Keywords:** Diclofenac, Bevacizumab, Diabetic Macular Edema, Intravitreal Injection, Anti-inflammatory Agents

## Abstract

The aim of this study was to compare the effect of intravitreal diclofenac, a non-steroidal anti-inflammatory drug (NSAID), with that of bevacizumab, a well-known anti-vascular endothelial growth factor (VEGF) drug, in the treatment of diabetic macular edema (DME). Diclofenac was chosen in this study because it has both features of NSAIDs and corticosteroids by inhibiting the cyclooxygenase (COX) and lipoxygenase pathways, respectively. In this non-randomized comparative interventional case series, 64 eyes from 32 patients with bilateral naïve DME were selected and every eye was randomly assigned to intravitreal injection of bevacizumab (IVB) or diclofenac (IVD). After exclusion of some patients because of short follow-up duration or less than two intravitreal injections, finally, 52 eyes from 26 patients were analyzed. Of those, 26 eyes received 500 µg/0.1 mL IVD and 26 eyes received 1.25 mg IVB. After 6 months of follow-up, the results indicated that visual acuity was significantly improved from 0.50 ± 0.13 in IVB and 0.52 ± 0.12 LogMAR in IVD at baseline to 0.2 ± 0.1 and 0.29 ± 0.07, respectively. Central macular thickness (CMT) and macular volume were measured based on spectral-domain optical coherence tomography (OCT) at month 1, 3, and 6. Both groups showed a significant reduction in CMT and macular volume from baseline but there was no significant difference between the IVB and IVD groups. Interestingly, IVD, but not IVB, decreased intraocular pressure (IOP), which is a desirable effect. There was no serious complication due to injections. This study sheds light into the long-term effects of NSAIDs and may support the idea that inflammation suppression by NSAIDs may have the same results as anti-VEGF administration.

## INTRODUCTION

As diabetes mellitus progress, diabetic macular edema (DME) can develop at any time of disease. DME represents one of the most important causes of vision loss in patients with diabetes [1]. There are different treatment strategies for DME. Recently, intravitreal injection of anti-vascular endothelial growth factor (VEGF) drugs has become very popular. Many studies have investigated the effect of ranibizumab and bevacizumab on diabetic retinopathy, especially DME [2, 3]. Corticosteroids are another class of well-known agents used in intravitreal injections in DME cases [4]. DME is the result of different pathophysiological pathways including angiogenesis and prostaglandin-induced inflammation [5, 6]. Anti-angiogenic agents such as ranibizumab and bevacizumab are used to inhibit VEGF, and triamcinolone is used to suppress prostaglandin-induced inflammation. Non-steroidal anti-inflammatory drugs (NSAIDs) inhibit the biosynthesis of prostaglandins and have emerged as a new possible treatment for DME [7, 8]. The inflammatory response responsible for initiation of DME causes the release of arachidonic acid from cell membrane phospholipids. Arachidonic acid turns into prostaglandins (PGs) and thromboxanes by means of cyclooxygenase enzymes (COX1 and COX2) and into leukotrienes by means of 5-lypoxygenase enzymes. The interesting point about prostaglandins is their potency for induction of angiogenesis. One study on cultured Müller cells showed that PGs induce the expression of VEGF. NSAIDs, which are potent COX inhibitors and anti-inflammatory agents, can have antiproliferative and antiangiogenic effects as well [9]. Another mechanism that may explain the antiangiogenic properties of NSAIDs is the inhibition of COX, which in turn reduces PGE1 and PGE2 production. This effect can induce vascular regression in chronic granulomatous inflammation [10, 11]. Although corticosteroids are thought to potently reduce inflammation by inhibiting two pathways simultaneously (the COX and 5-lypoxygenase pathways), NSAIDs have less adverse side effects than corticosteroids. These side effects include increased intraocular pressure (IOP) and cataract formation in patients with diabetics who are more vulnerable to increased IOP and cataract formation because of glucose fluctuations [12, 13]. On the other hand, injection of corticosteroids needs to be repeated quickly because of the short-term outcomes, which in turn increases the chance of adverse side effects [14, 15]. Another desirable characteristic of NSAIDs is tumor necrosis factor (TNF)-alpha suppression, which may inhibit early diabetic retinopathy progression [7]. Topical usage of NSAIDs is limited by inappropriate delivery to the posterior segment because of the blood–aqueous barrier. Intravitreal delivery of NSAIDs could enhance drug concentration in the posterior segment, especially in inflammatory diseases such as DME and age-related macular degeneration (AMD) [16]. Diclofenac sodium in a member of the NSAID family that can inhibit both the COX and lipoxygenase pathways, making it as potent an agent as any corticosteroid, with the advantage of not having those unwanted side effects [17]. Intravitreal injection of diclofenac sodium (IVD) is effective in inflammatory retinal diseases such as retinal vein occlusions, AMD, uveitic CME, and DME [18, 19]. There are a limited number of studies on IVD effects in DME. However, recently, the number of studies has grown and a small, randomized trial in patients with DME disclosed that IVD has similar therapeutic outcomes to intravitreal triamcinolone injections [17]. Ketorolac is another member of the NSAID family that has promising results in the treatment of DME, as reported in some case series [20, 21]. The aim of this study was to compare the visual and anatomical outcomes of IVD with those of intravitreal injection of bevacizumab (IVB). IVD is a safer choice for intravitreal injections. This study aimed to investigate this effect in patients with DME who were followed up in the long term.

## MATERIALS AND METHODS

This non-randomized comparative interventional case series was conducted in Tehran University of Medical Sciences from December 2015 to May 2016. Ethical approval number by review board/ethics committee of the Ophthalmic Research Center of Tehran University of Medical Sciences received (Rc-9305-23). All subjects who participated in the study signed an informed consent form before beginning the study. A separate informed consent form about probable complications of intravitreal injections was signed by all patients before entering the study. Sixty-four eyes from 32 patients with bilateral naïve DME were enrolled in the study. The inclusion criteria were based on the Early Treatment Diabetic Retinopathy Study (ETDRS) definition of clinically significant macular edema with foveal involvement [22]. Exclusion criteria were any prior panretinal or focal laser photocoagulation, intraocular surgery or any type of intraocular injections before this study, glaucoma, visual acuity (VA) of 20/40 or better, VA of 20/200 or worse, iris neovascularization, vitreomacular traction or uveitis, proliferative diabetic retinopathy, severe ischemic maculopathy (large irregular foveal avascular zones on fluorescein angiography with distinct areas of capillary nonperfusion within 1 disc diameter of the foveal center), any other cause of macular edema, significant media opacity, and any other associated macular diseases. The other exclusion criteria were a total follow-up of less than 6 months and a total number of intravitreal injections less than 2 in 6 months of follow-up, which resulted in exclusion of six patients. At the end of the study, 52 eyes from 26 patients were analyzed. All the patients underwent baseline blood testing to determine the metabolic control status including glycated hemoglobin (HbA1c), low-density lipoprotein (LDL), high-density lipoprotein (HDL), and triglyceride (TG). All included eyes underwent a complete baseline ophthalmic examination including best-corrected visual acuity (BCVA), IOP evaluation, slit-lamp biomicroscopy, and fundus photography. Measurement of central macular thickness (CMT), central macular volume, and macular volume was performed by using spectral-domain optical coherence tomography (OCT) (Heidelberg Engineering, Vista, California, USA). BCVA was evaluated by Snellen charts and was recorded in the logarithm of the minimum angle of resolution (logMAR) scale. Each eye of a patient was randomly assigned to one of two study groups: in the IVD group, each eye received 500 µg/0.1 mL of diclofenac sodium (Troge Medical GMBH, Hamburg, Germany) diluted with balanced salt solution intravitreally with a 27-gauge needle through the superotemporal quadrant; in the IVB group, each eye received 1.25 mg/0.05 mL of bevacizumab (Avastin; Genentech Inc., South San Francisco, CA, USA (made for F. Haffmann-La Roche, Ltd., Basel, Switzerland)), which was injected with through the superotemporal quadrant with a 27-gauge needle. All injections were performed under sterile conditions using Betadine 5% (applied two times, separated by 5 minutes) and anesthetic eye drops (two times, 3 minutes apart) with insertion of a lid speculum. The study drugs were injected at baseline and then every 1 to 1.5 months unless visual acuity was 20/20 or there was no improvement or worsening in response to the past two injections. Two examinations were done for each eye to check mostly the anterior chamber reaction and IOP rise on day 1 and 7 after the first injections. Complete ophthalmologic examination was performed at months 1, 3, and 6 after intervention. OCT was repeated at months 1, 3, and 6 and central retinal thickness was measured in a circle of 3 mm in diameter centered on the fixation point. Macular volume was measured, too. The primary outcome measure was the change in best-corrected logMAR. CMT changes and central macular volume as reported in OCT prints were the secondary outcome measures. No severe injection-related complication was observed during the study period. Ocular inflammation other than endophthalmitis that was in form of anterior chamber reaction was observed in two eyes in the IVB group and one eye in the IVD group, which was controlled by topical corticosteroids. All procedures were run by staff members other than the study investigators. Skilled examiners measured refraction and BCVA and performed OCT at baseline and at each study visit. The examiners were masked both to the randomization and to the findings of earlier measurements. Patients and investigators were not informed of randomization and details of IVB and IVD treatment data.

Statistical analysis was performed with SPSS software (version 19; SPSS, Inc., Chicago, IL). Mean ± standard deviation (SD), median (interquartile range), range, and frequency (percent) were used for descriptive purposes. Paired samples t-test and Wilcoxon test were done for comparing the values with the baseline within each treatment group. Mann–Whitney and paired t-test were used to compare variables between groups. Analysis of covariance (ANCOVA) and ordinal logistic regression was used to adjust for the baseline differences. P values less than 0.05 were considered as statistically significant

## RESULTS

Fifty-two eyes (26 right eyes and 26 left eyes) from 26 patients with bilateral DME were analyzed. The mean age of the patients was 60.1 years (range 53 to 66 years). Eighteen were men and 34 were women. Fifteen patients had moderate non-proliferative diabetic retinopathy (NPDR) and 37 patients had severe NPDR. Table 1 summarizes the baseline demographic and metabolic control characteristics of both groups at baseline. Table 2 compares visual acuity and macular OCT variables including CMT and macular volume between the IVB and IVD groups. No significant difference was detected between the two groups at baseline.

**Figure 1:**
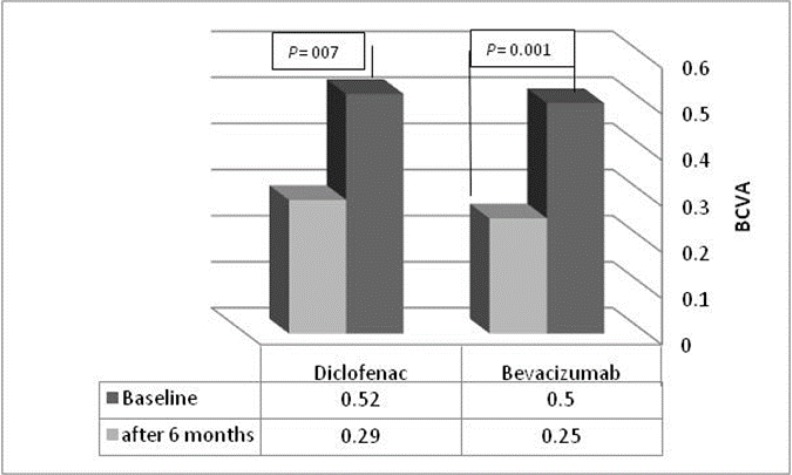
Change in Best-Corrected Visual Acuity (BCVA) after 6 Months in Each Group

Before intervention, the BCVA in the IVB group was 0.50 ± 0.13 logMAR. The BCVA in the IVD group was 0.52 ± 0.10 logMAR and the difference between groups was not statistically significant (P = 0.540). After 6 months follow up, the BCVA improved significantly in both groups (Fig 1); it was 0.2 ± 0.1 in the IVB group and 0.29 ± 0.07 in the IVD group, which was statistically significant based on t-test. At 1, 3, and 6 months of follow-up, there was no significant difference between BCVA of the IVB and IVD groups (P > 0.05) (Table 3).

**Figure 2:**
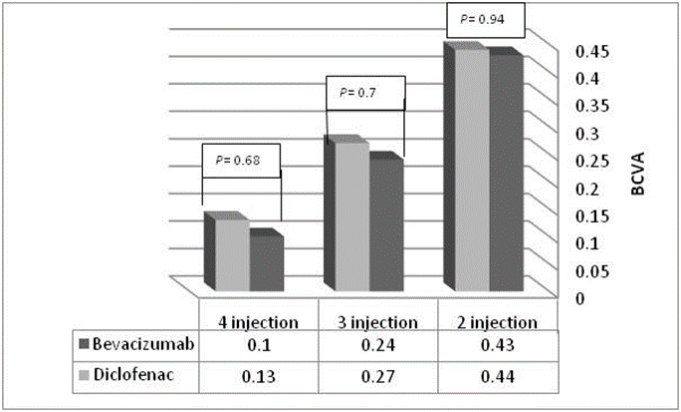
Comparison of Best-Corrected Visual Acuity (BCVA) at 6 Months of Follow-Up between the Two Groups based on the Number of Injections.

At 6 months of follow-up, 12 eyes (23.1%) had received two injections, 32 eyes (61.5%) had received three injections, and eight eyes (15.4%) had received four injections; there was no significant difference between the IVB and IVD groups (Fig 2).

To examine the role of baseline variables on final BCVA, we performed a multivariate linear regression analysis (Table 4).

**Table 1:** Demographics and Clinical Data of the Enrolled Patients

Variable	Value
Age (years)	60.1 ± 6.7
Sex	
** Male**	18 (34.6%)
** Female**	34 (65.4%)
Diabetic retinopathy stage	
** Moderate NPDR**	15 (28.8%)
** Severe NPDR**	37 (71.2%)
Metabolic control features	
** HbA1c (mg/dL)**	7.8 ± 1.2
** LDL (mg/dL)**	121.3 ± 23.9
** HDL (mg/dL)**	40.1 ± 7.4
** TG (mg/dL)**	181.8 ± 82.1

Data in table are presented as mean ± SD or No (%). LDL (low density lipoprotein), HDL (high density lipoprotein), TG (triglyceride)

**Table 2:** Comparison of Visual Acuity and Macular Optical Coherence Tomography (OCT) Variables between the Two Groups

Variable	IVB (n = 26)	IVD (n = 26)	p-value*
BCVA (logMAR)	0.50 ± 0.13	0.52 ± 10	0.540
Central macular thickness (µm)	416.8 ± 116.2	439.2 ± 94.4	0.533
Macular volume (mm^3^)	10.8 ± 1.23	10.91 ± 1.41	0.411
Central macular volume (mm^3^)	0.34 ± 0.09	0.37 ± 0.12	0.736

* Based on a t-test, IVB = intravitreal bevacizumab, IVD = intravitreal diclofenac, logMAR = logarithm of the minimum angle of resolution, best-corrected visual acuity (BCVA)

**Table 3:** Comparison of Visual Acuity in Follow-up Visits between the Two Groups

Variables	IVB (n = 26)	IVD (n = 26)	P-value*
Baseline BCVA (logMAR)	0.50 ± 0.13	0.52 ± 10	0.540
BCVA at the 1-month follow-up (logMAR)	0.41 ± 0.12	0.44 ± 0.14	0.825
BCVA at the 3-month follow-up (logMAR)	0.28 ± 0.0.9	0.31 ± 0.13	0.466
BCVA at the 6-month follow-up (logMAR)	0.25 ± 0.1	0.29 ± 0.07	0.442

* Based on a t-test; IVB = intravitreal bevacizumab, IVD = intravitreal diclofenac, logMAR = logarithm of the minimum angle of resolution, best-corrected visual acuity (BCVA)

**Table 4:** Multivariate Linear Regression Analysis

Variable	Odds ratio (95% confidence interval)	p value
Number of injections	0.158 (0.17, 0.299)	0.029
Baseline central macular thickness (µm)	0.0 (0.0, 0.001)	0.653
Reduction in central macular thickness after the first injection (µm)	0.002 (0.0, 0.004)	0.017
Stage of diabetic retinopathy (severe NPDR)	0.102 (-0.51, 255)	0.181
HbA1c (mg/dL)	-0.96 (-0.168, -0.24)	0.011

The intravitreal bevacizumab (IVB) group was the reference parameter and the odds ratio of the intravitreal diclofenac (IVD) group was evaluated based on the IVB group. This analysis shows the effect of variables on final best-corrected visual acuity (BCVA)

The number of injections and the reduction in CMT after the first injection (µm) and baseline HbA1c (mg/dL) were factors that had a significant impact on final BCVA.

**Macular Thickness and Volume**

The mean baseline CMT in the IVB group was 409.8 ± 116.8 µm and 428.2 ± 94.4 µm in the IVD group. There was no significant difference between the two groups (P = 0.533). After 1 month, the CMT decreased to 386.1 ± 101.2 µm in the IVB group and 406.1 ± 92.5 µm in the IVD group. After 3 months, the CMT was 355.4 ± 95.1 µm in the IVB group and 355.4 ± 95.1 µm in the IVD group. After 6 months, it was 348.4 ± 98.1 µm in the IVB group and 360.7 ± 83.1 µm in the IVD group. There was no statistically significant difference between the two groups at any time point (Table 5).

**Figure 3:**
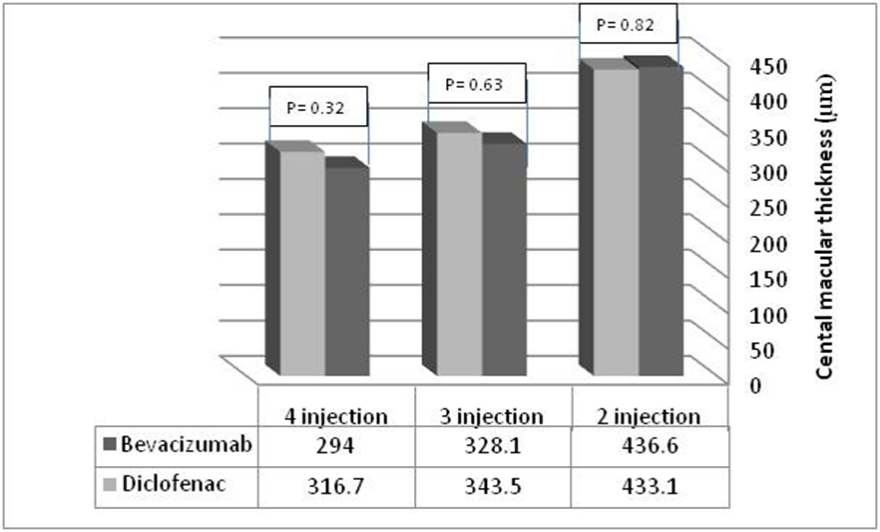
Comparison of Central Macular Thickness at 6 Months of Follow-up between the Two Groups based on Number of Injections

At 6 months, the macular thickness change to baseline thickness was -59.6 ± 99.4 in the IVB group and -66.1 ± 79.6 in the IVD group (P = 0.06 for the IVB groups and P = 0.00 for the IVD group) (Table 5). Macular volume at baseline was 10.8 ± 1.23 mm^3^ in the IVB group and 10.91 ± 1.41 mm^3^ in the IVD group, which was reduced to 10.17 ± 1.04 mm^3^ and 10.38 ± 1.47 mm^3^, respectively, after 6 months. Macular volume change from baseline to 6 months was -0.53 ± 0.75 in the IVB group and -0.44 ± 0.76 in the IVD group (P = 0.008 for the IVB group and P = 0.002 for the IVD group) (Table 6). There was no significant difference between macular volumes in the two groups. The number of injections did not significantly affect macular thickness changes in both groups (Fig 3, Table 6).

The IOP was measured in both groups at baseline and 1 week after injection. The IOP was reduced in the IVD group from 16.1 mmHg to 14.3 mmHg (P = 0.013) (Table 7).

The IOP increased in the IVB group from 15.7 mmHg to 16/4 mmHg but it was not statistically significant (P = 0.3). IVD may reduce the IOP, which is a desirable effect for patients with diabetes who may have increased IOP due to many reasons.

**Table 5:** Comparison of Central Macular Thickness between the Two Groups in Follow-up Visits

Variables	IVB (n = 26)	IVD (n = 26)	P-value*
Central macular thickness (µm)	416.8 ± 116.2	439.2 ± 94.4	0.533
Central macular thickness at the 1-month follow-up (µm)	386.1 ± 101.2	406.1 ± 92.5	0.740
Central macular thickness at the 3-month follow-up (µm)	355.4 ± 95.1	394.9 ± 87.8	0.462
Central macular thickness at the 6-month follow-up (µm)	348.4 ± 98.1	360.9 ± 83.1	0.648
Central macular thickness change at month 6 compared to baseline central macular thickness	-59.6 ± 99.4	-66.1 ± 79.6	
P-value among groups **	0.06	0.00	

* Based on a t-test,

** based on a paired t-test, IVB = intravitreal bevacizumab, IVD = intravitreal diclofenac

**Table 6:** Comparison of Macular Volume between the Two Groups in Follow-Up

Variables	IVB (n = 26)	IVD (n = 26)	P-value*
Macular volume (mm^3^)	10.8 ± 1.23	10.91 ± 1.41	0.411
Macular volume at the 1-month follow-up (mm^3^)	10.63 ± 1.2	10.75 ± 1.65	0.853
Macular volume at the 3-month follow-up (mm^3^)	10.22 ± 0.92	10.51 ± 1.61	0.606
Macular volume at the 6-month follow-up (mm^3^)	10.17 ± 1.04	10.38 ± 1.47	0.621
Macular volume change at month 6 compared to baseline macular volume	-0.53 ± 0.75	-0.44 ± 0.76	
p-value within **	0.002	0.008	

* Based on a t-test,

** based on a paired t-test, IVB = intravitreal bevacizumab, IVD = intravitreal diclofenac

**Table 7:** Comparison of Intraocular Pressure (IOP) between Baseline and Following the First Injection in Each Group

Study group	Baseline	1 week after injection	p-value*
IVD	16.1 ± 3.1 mmHg	14.3 ± 2.7 mmHg	0.031
IVB	15.7 ± 2.9 mmHg	16.4 ± 4.1 mmHg	0.312

* Based on a paired t-test, IVB = intravitreal bevacizumab, IVD = intravitreal diclofenac

## DISCUSSION

Intravitreal injections are being progressively used for the treatment of diabetic retinopathy, especially DME. Several studies have shown their superiority to traditional methods [23]. There are different intravitreal drugs that can be used to stop the progression of diabetic retinopathy. It is important to understand the underlying mechanism of diabetic retinopathy to choose the proper drug. Many studies support the idea of using anti-VEGF treatments for DME [3, 24]. The other rational theory to stop the inflammation pathway involves corticosteroids, which have been used for the treatment of DME [25-27]. Corticosteroids are capable of inhibiting both COX and 5-lypoxygenase pathways but have severe complications such as cataract formation and increased IOP [13, 14]. In this non-randomized comparative interventional case series, we chose NSAIDs. Another study has supported the unique ability of diclofenac to stop the lipoxygenase pathway. This unique quality of diclofenac makes it similar to corticosteroids and a potent member of the anti-inflammatory NSAIDs [28]. In previous studies, different types of NSAIDs have been used for the treatment of DME. For example, topical nepafenac has been shown to be effective for DME treatment [29]. After cataract surgery, topical bromofenac was also suitable for inhibition of cystoids macular edema (CME) in patients with diabetes [30]. In this study, we examined the intravitreal route of administration because of better penetration of NSAIDs to the posterior segment. Direct injection into the vitreous can enhance the therapeutic effect of drug because of longer drug entrapment in the vitreous, which prolongs the half-life of the drug. The results indicated that both anti-VEGF and NSAIDs improved visual acuity and decreased macular thickness and macular volume after intravitreal injection significantly. However, there was no statistically significant difference between the two groups. On the other hand, NSAIDs lowered the IOP after 1 week but anti-VEGF increased IOP during the same period. This rise of IOP was not significant. This study had a longer follow-up of patients and the results support those of previous studies by Soheilian et al., who showed visual improvement in DME and other causes of macular edema [18, 31]. Elbendary et al. also confirmed the beneficial use of intravitreal diclofenac in comparison to corticosteroids on macular edema in patients with diabetes by visual acuity improvement and macular thickness changes. They also showed that NSAIDs reduce IOP in IVD-treated patients [17]. Intravitreal injection of diclofenac has been useful in refractory diabetic macular edema, too [20, 21]. In patients with diabetes, topical diclofenac was able to decrease IOP after cataract surgery [32]. This effect was the same in diabetic retinopathy patients after intravitreal injection. The long-term follow-up in this study is another strong point, which has overcome the limitation of previous studies with short sample size and short follow-up time because of the short half-life of IVD (only 2.87 h) [33]. In 2016, it was shown that combination of intravitreal diclofenac with intravitreal bevacizumab was superior to bevacizumab alone [34]. Intravitreal diclofenac is effective and safe for improving BCVA and decreasing central macular thickness in patients who had vascular accident of branch retinal vein occlusion (BRVO) with macular edema [35]. The repeated injection of intravitreal diclofenac has helped overcome the problem of short half-life. A 6-month follow-up can somehow ensure that NSAIDs are reliable in the long term, too. Anti-VEGF drugs, the current choice for patients with DME, may be replaced by NSAIDs, which can improve the BCVA and decrease CMT at the same level, with the additional effect of IOP lowering. In the future, clinical trials longer follow-up periods with larger sample sizes will help decide whether NSAIDs can replace anti-VEGF drugs or be combined with other drugs in order to compensate our limitations. Future studies should address whether NSAIDs can decrease the need for repeated injections. The reduction in number of intravitreal injections is a very important outcome for patients that could enhance patient compliance with the therapy for diabetic retinopathy. For more precise comparison of IVB and IVD, a randomized clinical trial may be desirable.
